# On the detection of functionally coherent groups of protein domains with an extension to protein annotation

**DOI:** 10.1186/1471-2105-8-390

**Published:** 2007-10-16

**Authors:** William A McLaughlin, Ken Chen, Tingjun Hou, Wei Wang

**Affiliations:** 1 Department of Chemistry and Biochemistry, Center for Theoretical Biological Physics, University of California, San Diego, 9500 Gilman Drive La Jolla, CA 92093-0359, USA

## Abstract

**Background:**

Protein domains coordinate to perform multifaceted cellular functions, and domain combinations serve as the functional building blocks of the cell. The available methods to identify functional domain combinations are limited in their scope, e.g. to the identification of combinations falling within individual proteins or within specific regions in a translated genome. Further effort is needed to identify groups of domains that span across two or more proteins and are linked by a cooperative function. Such functional domain combinations can be useful for protein annotation.

**Results:**

Using a new computational method, we have identified 114 groups of domains, referred to as domain assembly units (DASSEM units), in the proteome of budding yeast *Saccharomyces cerevisiae*. The units participate in many important cellular processes such as transcription regulation, translation initiation, and mRNA splicing. Within the units the domains were found to function in a cooperative manner; and each domain contributed to a different aspect of the unit's overall function. The member domains of DASSEM units were found to be significantly enriched among proteins contained in transcription modules, defined as genes sharing similar expression profiles and presumably similar functions. The observation further confirmed the functional coherence of DASSEM units. The functional linkages of units were found in both functionally characterized and uncharacterized proteins, which enabled the assessment of protein function based on domain composition.

**Conclusion:**

A new computational method was developed to identify groups of domains that are linked by a common function in the proteome of *Saccharomyces cerevisiae*. These groups can either lie within individual proteins or span across different proteins. We propose that the functional linkages among the domains within the DASSEM units can be used as a non-homology based tool to annotate uncharacterized proteins.

## Background

Protein domains are sequential, structural, and functional units [1]. They perform and regulate catalysis, provide structural building blocks, and/or act as interaction mediators that link together cellular pathways. Protein domains can also be combined together to perform multifaceted functions [2-6]. For example, a DNA-binding domain can be combined with a dimerization domain to allow for cooperative DNA-binding [7]; and the SH2, SH3, and kinase domains can be combined to facilitate signal transduction [8]. A protein can be better characterized by the function of its domain combination rather than the functions of its individual domains. That deduction has been corroborated by the observation that function is better conserved across multi-domain proteins than across single domain proteins [9].

There are a variety of methods available for the identification of functional domain combinations based on protein sequence information, and these methods vary in both the scope of the combinations identified and in their applications. As examples, domain combinations can be identified by finding domain fusion pairs [10,11], prevalently co-occurring protein domains within individual protein sequences [12], densely interconnected domains within the protein domain networks [6,13], and domains that co-occur along particular stretches of the translated genome [14]. The methods can therefore be limited to the identification of combinations that occur within individual proteins, within a densely linked domain network, or within particular genomic regions (see Discussion for details). Further effort is needed to automatically identify groups of domains that perform particular cellular functions and automatically provide annotation to these groups.

For the present study, a systematic method was developed to automatically identify functionally coherent groups of protein domains, referred to as domain assembly units or DASSEM units, and their corresponding functions. The method employed a soft-margin clustering technique that was guided by singular value decomposition (SVD). SVD is often used to capture the significant variance in a large dataset, and here it was used to retrieve the highly prevalent domain combinations found in an adjacency matrix of proteins versus domains. The prevalent domain combinations were clustered such that, when necessary, a domain was assigned to multiple groups in order to reflect the fact it can participate in different functions. Note that the clustering method is similar in spirit to the fuzzy k-means clustering method used for the extraction of coherent expression patterns from microarray experiments [15].

The current method was applied to the protein/domain complement of *Saccharomyces cerevisiae*, and 114 functionally coherent groups of domains, referred to as domain assembly units (DASSEM units), were identified. The functions of the units included a broad range of cellular tasks such as chromatin modification, carbohydrate transport, translation, and ubiquitin-dependent protein catabolism. Within the units, the functional linkages among the domains were demonstrated in three ways. First, there was a significant enrichment of Gene Ontology (GO) terms in proteins contributing domains to the units, which suggested that the domains were used in a functionally coherent manner. Second, the domains of DASSEM units were shown by manual review to be utilized in a rational way to facilitate particular cellular processes. Third, DASSEM units overlapped significantly with transcription modules, defined as groups of genes that share the same expression pattern under a set of cellular conditions. Such overlap further confirmed the functional coherence of DASSEM units.

We found that the functional linkages within DASSEM units can allow for the prediction of protein function based on domain composition. Since the transfer of annotation from a DASSEM unit to a protein of unknown function does not require high sequence homology between the unknown protein and an annotated one [16], the method can be regarded as a non-homology-based method for functional annotation [17,18]. Non-homology based methods for protein functional annotation include phylogenetic profiling [19-21], chromosome proximity [22,23], text mining [24,25], domain fusion pairs analysis [11,26,27], and combination of these predictors such as mRNA co-expression and phylogenetic profiling [28]. Databases and annotation tools such as Prolinks [29], STRING [30], and Predictome provide an consolidation of methods for predicting the function of a protein using non-homology-based methods [26]. These methods have the property that a protein of unknown function can be annotated based on its biological context. i.e. how it relates to proteins of known function [16,31]. In a conceptually similar way, the DASSEM unit provides functional annotation by placing a protein in the context of domain combination that has been functionally characterized. DASSEM units thereby provide an additional means to predict protein function. Also, the annotation of DASSEM units can partially overcome the inaccuracy or incomplete annotation of individual domains.

## Results

### Derivation of domain assembly units

A domain assembly unit can be viewed as a group of domains that are linked together by domain fusion events or events that cause domains that function together to be placed in the same protein. For example, if domain A is fused with domain B in one protein while domain B is fused with domain C in a second protein, domains A and C can be functionally linked. If multiple instances are found where A is fused to B and B is fused to C, the functional inference between A and C can be strengthened, and as a consequence the two proteins containing domains A and C can be found to participate in the same biological process (see below for examples). The cycle can continue with more domain fusions and lead to a larger group of functionally linked domains/proteins. These groups are required for higher order cellular functions.

**Figure 1:**
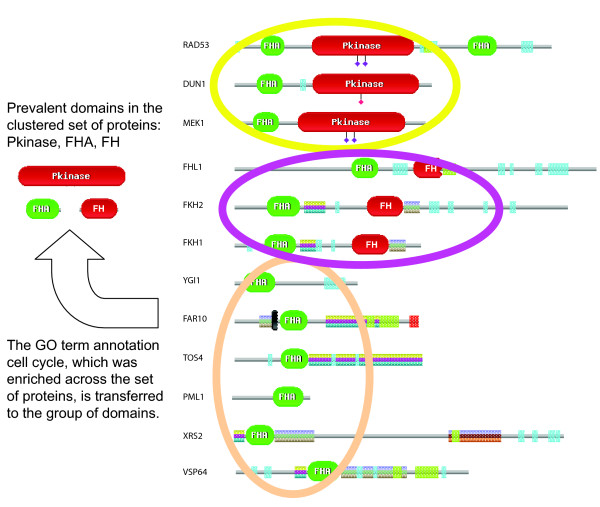
**An illustration of the derivation of a DASSEM unit and its functional annotation**. For the group of proteins shown, there are three prevalent domain compositions within individual proteins (circled). The domain fusions of these prevalent domain compositions were used to link three domains and to create a DASSEM unit that contains the fork head (FH) domain, the fork head associated (FHA) domain and the kinase domain. The overall function of the DASSEM unit was obtained by finding the GO terms that were enriched across the proteins associated with the unit. The GO term enrichment indicated that the unit participates in the cell cycle. Schematics of domains and proteins were taken from the Pfam database [89].

Shown in Figure 1 is an illustration of how an example DASSEM unit was identified and annotated. The first step was to find an initial set of domain combinations that are prevalent in the adjacency matrix of proteins versus domains, which are circled. These prevalent domain combinations are then spliced together through a series of domain fusions. In the example, three domains were pieced together by two different domain fusion events. FHA was found fused to FH and also to the kinase domain, and these two domain fusions forged a functional linkage between the FH and kinase domains. The three domains constituted a DASSEM unit, and proteins were associated with the unit based on their domain composition (see Methods for details). The function of the unit was derived by identifying the GO terms that were enriched across all of the proteins associated with the unit using the GO::TermFinder tool [32].

We derived 114 DASSEM units from the domain content of protein sequences within the proteome of *Saccharomyces cerevisiae*. Shown in Table 1 are six example units; and full lists of the units along with all their functional annotation are available online in the supplementary material [33]. The number of domains per unit ranged from 1 to 11, and the average was 3.3. Twenty six of the units contained only one domain. These domains had a high prevalence in proteins but a relatively low degree of co-occurrence with other domains. Although technically not combinations, the annotation of these domains can provide insight into their function. An example is the DASSEM unit which consists of the amino acid permease domain [PFAM:PF00324]. All 22 proteins that contributed domains to the unit contain only this one domain, which implies that the domain may not require a cooperation with any other domain or it may be too large (~500 amino acids) to be fused with other domains within a protein sequence.

The functional annotation for each DASSEM unit was automatically generated by finding the Gene Ontology (GO) terms that were enriched (p-value < 0.05) across the proteins associated with the unit (Figure 1) [32]. In order to account for the fact that multiple tests were performed, the Bonferroni correction was used when the p-values were calculated [32,34]. All of the 114 DASSEM units were found to have significantly enriched GO terms; and the median top p-values of the annotations across all the units was 7.7*10^-7 ^for the GO term biological process category, 4.1*10^-03 ^for the component category, 9.9*10^-13 ^for the molecular function category (see example DASSEM units in Table 1).

**Table 1:** Six example domain assembly units and their corresponding annotations.

**DASSEM units**	**Gene Ontology term annotation**
**1**.	
FHA domain (PF00498)	P-cell cycle 4.32*10^-06^
Fork head domain (PF00250)	C-nucleus 2.12*10^-02^
Protein kinase domain (PF00069)	F-transcription factor activity 4.22*10^-05^
**2**.	
Fungal Zn(2)-Cys(6) domain (PF00172)	P-regulation of transcription: 8.33*10^-17^
Fungal transcription factor domain (PF04082)	C-nucleus, 5.48*10^-12^
Gal4-like dimerization domain (PF03902)	F-transcription regulator, 2.24*10^-26^
PAS fold domain (PF00989)	
**3**.	
ABC transporter domain (PF00005)	P-transport, 3.02*10^-05^
ABC transporter region 1 domain (PF00664)	C-membrane, 4.15*10^-05^
ABC transporter region 2 domain (PF06472)	F-ATPase activity, 5.68*10^-23^
ABC-2 type transporter (PF01061)	
Metal-binding domain in RNase L domain (PF04068)	
4Fe-4S binding domain (PF00037)	
**4**.	
GTPase of unknown function domain (PF01926)	P-ribosome-nucleus export, 1.51*10^-02^
DUF933 domain (PF06071)	C-mitochondrial inner membrane, 3.27*10^-02^
TGS domain (PF02824)	F-GTP binding, 9.86*10^-09^
GTP1/OBG domain (PF01018)	
**5**.	
Helicase conserved C-terminal domain (PF00271)	P-chromosome organization, 5.41*10^-06^
SNF2 family N-terminal domain (PF00176)	C-chromatin remodeling complex, 1.77*10^-03^
HSA (PF07529)	F-ATPase activity 2.04*10^-07^
Chromatin organisation modifier domain (PF00385)	
**6**.	
Elongation factor Tu GTP binding domain (PF00009)	P-translation factor activity 1.08*10^-20^
Elongation factor Tu domain 2 (PF03144)	C-ribosome 3.71*10^-04^
Elongation factor G C-terminus (PF00679)	F-translation 1.33*10^-09^
Elongation factor G, domain IV (PF03764)	
Elongation factor Tu C-terminal domain (PF03143)	
GTP-binding protein LepA C-terminus (PF06421)	
Translation initiation factor IF-2, N-term. (PF04760)	

Each domain assembly unit or DASSEM unit is described as a list of Pfam domains and its GO term annotation. The terms which have the most significant p-values for the GO categories of biological process (P), cellular component (C), and molecular function (F), are shown for each unit. The full lists of enriched GO terms are provided in a companion website [33].

### Cooperative nature of domains within DASSEM units

The domains within the DASSEM units cooperate with one another in order to achieve a particular function, which was made apparent through an examination of the functional roles of proteins associated with the units. Three example DASSEM units are described in following in order to show how such cooperation may occur.

The first example, DASSEM 1 in Table 1, participates in the cell cycle and consists of three domains: fork head associated (FHA) domain [PFAM:PF00498], the fork head domain [PFAM:PF00250], and the protein kinase domain [PFAM:PF00069]. The proteins associated with the unit are listed in Figure 1. The proteins Fkh1 and Fkh2 are transcription factors that regulate expression of cell cycle genes while Rad53 and Dun1 are cell cycle checkpoint regulators [35-37]. Transcription factor Fhl1 regulates the gene expressions of ribosomal proteins, and multiple genomic analyses have shown that it is also a cell cycle regulator [38,39]. Although Mek1 has not been shown to be involved in cell cycle, it is a serine/threonine protein kinase required for meiotic recombination and normal spore viability [40]. Among the rest of the proteins associated with the unit, as annotated in the *Saccharomyces *Genome Database (SGD) [41,42], most of them also play a role the cell cycle. Tos4 is a transcription factor that regulates genes expressed in G1/S phase of cell cycle. Far9 and Far10 are involved in pheromone induced cell cycle arrest. Pml1 is required for nuclear retention of unspliced pre-mRNAs. The YGL081W open reading frame is a hypothetical gene with unknown functions. If YGL081W is confirmed to be a real gene, it is reasonable to predict that it is involved in cell cycle based on the DASSEM unit's function.

With regard to the cooperation of the domains of the unit, they have been modeled to control the cell cycle progression through the G2/M phase [43]. Specifically the model proposed that the phosphorylation of the protein Ndd1 by Cbl kinase allows it to bind to the FHA domain of the protein Fkh2. The binding of Ndd1 facilitates an active transcriptional complex formed between Ndd1, Fkh2 (which also contains the fork head DNA-binding domain), and the protein MCM1. The model has been confirmed by in a detailed study using phosphylation, binding, and transcription assays [44]. Note that as shown in Figure 1 the three domains of the DASSEM unit are not all contained within a single protein, so the combination can not be found by examining combinations that only occur within individual protein sequences. Also, because the protein kinase domain does not co-occur with FH domain, a domain network clustering analysis did not identify this domain combination since there is a low clustering coefficient [6,13].

A second DASSEM unit example is involved in the process of transcription regulation (p-value 8.13 × 10^-17^) and consists of four domains: the Fungal Zn(2)-Cys(6) binuclear cluster domain [PFAM:PF00172], the Fungal specific transcription factor domain [PFAM:PF04082], the Gal4-like dimerization domain [PFAM:PF03902], and the PAS domain [PFAM:PF00989]. The first three domains are the components of the GAL4 family transcription factors [45]. The binuclear cluster domain is relatively small and binds zinc to provide high structural stability for DNA-recognition [46]. The transcription factor domain binds DNA in a sequence specific manner [47], and the dimerization domain provides an interface for dimerization via a leucine zipper so that two proteins can bind to DNA cooperatively [7]. The PAS domain is involved in sensing stimuli such as the redox state of the cell [48], and can regulate transcription factor activity by facilitating dimerization [49]. The DASSEM unit implies a model of how the PAS domain can regulate transcription: a conformational change in the PAS domain induced by a change in redox state of the cell can allow the PAS domain to facilitate dimerization of GAL4 like transcription factors, which promotes the transcription of target genes.

A third example DASSEM unit combines domains of the ABC transporter domain cassette with the 4Fe-4S binding domain [PFAM:PF00037] and the metal-binding RNase L inhibitor domain [PFAM:PF04068]. The domains of ABC transporter cassette hydrolyzes ATP to facilitate the active transport of allocrites (ions or small molecules) against their concentration gradient through cellular membranes [50]. When the cassette is used in conjunction with the 4Fe-4S binding domain, it transports iron into the cell for the assembly of the 4Fe-4S cluster within the domain [51]. One of the functions of the 4Fe-4S cluster domain is to detect oxidatively damaged DNA [52,53], and when it is combined with the RNase L inhibitor domain, a role in DNA/RNA metabolism has been proposed [54]. The DASSEM unit pieces together a mechanism of iron transport and DNA repair: iron transport by an ABC transporter cassette allows for the assembly of the 4Fe-4S cluster which in turn lends DNA-binding capability to proteins involved in oxidative repair of DNA.

### DASSEM units are utilized in transcription modules

To further demonstrate the functional linkages among the domains within the DASSEM units, the units were shown to be utilized within transcription modules, defined as groups of genes that share the same expression pattern under a particular set of conditions and presumably have coherent functions [55,56].

**Figure 2:**
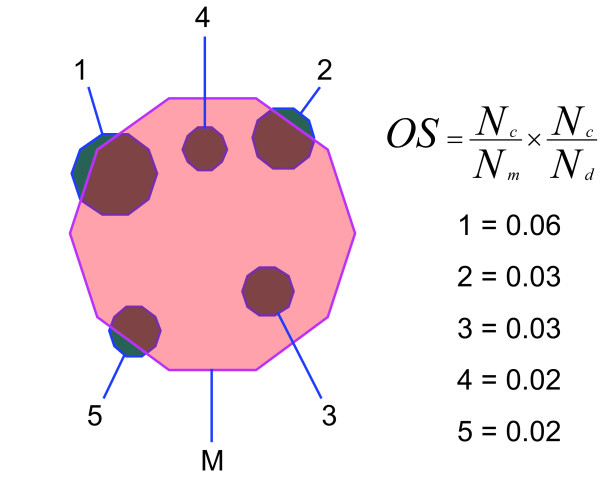
**An illustration of the utilization of DASSEM units within a transcription module**. The example transcription module is involved in the process of amino acid biosynthesis, and the DASSEM units contribute to necessary auxiliary processes. The terms listed are from the GO term "biological process" category. M- a transcription module involved in amino acid biosynthesis, 1- a unit involved in aromatic carbon metabolism, 2- a unit involved in sulfate assimilation, 3- a unit involved in serine biosynthesis, 4- a unit involved in ethanol metabolism, and 5- a unit involved in amino acid derivative metabolism. The equation for the overlap score is given along with the overlap scores for the DASSEM units in the example.

For each of the 86 transcription modules defined by Ihmels et al., we first identified DASSEM units that contained domains also present in the module. A Venn diagram shows an example transcription module with the DASSEM units that had the highest overlap scores (Figure 2) [57]. The diagram illustrates that the transcription module engaged multiple DASSEM units to carry out the process of amino acid biosynthesis, the primary biological process associated with the module according to the GO::TermFinder tool [32]. The DASSEM units that overlap with the module designate sub-processes that are required for the overall process of amino acid biosynthesis. Unit 1 participates in aromatic carbon metabolism, which is necessary for the synthesis of the aromatic amino acids phenylalanine, tryptophan, and tyrosine. Unit 2 participates in sulfate assimilation, which is needed to synthesize the amino acids methionine and cysteine. Unit 3 participates in serine family amino acid biosynthesis. Unit 4 participates in ethanol metabolism which produces pyruvate, a substrate used to make alanine. Unit 5 participates in the process of amino acid and derivative metabolism. The example indicates that DASSEM units are part of a hierarchy of function where domains combine to perform the function of a DASSEM unit; and DASSEM units are utilized to perform the subordinate functions of transcription modules. DASSEM units are therefore functional parts, albeit sometimes small functional parts, of the domain combinations found in transcription modules. Note that the overlap score, as depicted in Figure 2, is the fraction of domains in the module that are in common with the DASSEM unit multiplied by the fraction of domains in the DASSEM unit that are in common with the module, which can more simply referred to as the recall multiplied by precision. For unit 1 in the example these values are 0.072 (recall) and 0.818 (precision). Recall is usually very low as DASSEM units are parts of modules.

**Figure 3:**
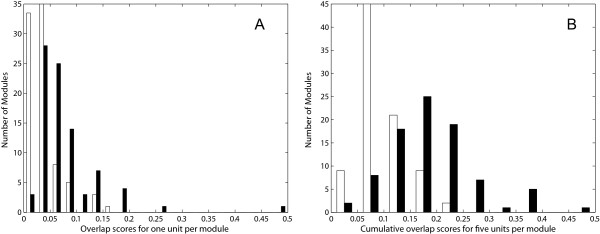
**Plots of the overlap scores of doma in content of DASSEM units with that of transcription modules**. Overlap scores of the DASSEM units with transcription modules are given for before (black) and after (white) randomization of the domains in the modules. The highest overlap scores, where one DASSEM unit was paired with each transcription module, are shown in panel A. The overlap scores were also calculated when a collection of five DASSEM used were paired with each transcription module. These are shown are shown in panel B. The plots indicate the DASSEM units were utilized in transcription modules, based their overlap of domain content.

To further demonstrate the overlap between of the DASSEM units with the transcription modules, the DASSEM units with the highest overlap scores with the transcription modules were examined. The distribution of the overlap scores for the highest overlapping DASSEM units with the modules is shown in Figure 3, panel A and the collective overlap of the highest five are shown in panel B. Overlap scores between the DASSEM units and the randomized modules are also shown. These randomized modules were generated by randomly redistributing domains across the 86 transcription modules.

**Figure 4:**
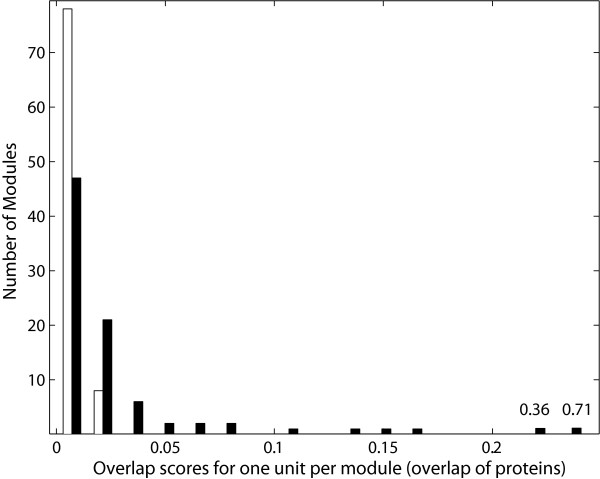
**Plot of the overlap scores of protein content of DASSEM units with that of transcription modules**. The overlap scores of the DASSEM units with transcription modules are given for before (black) and after (white) randomization of the **proteins **in the modules. The highest overlap scores, where one DASSEM unit was paired with each transcription module, are shown. Student *t*-tests were used to compare the average overlap scores of the DASSEM unit with the original versus the randomized modules. The p-value of a Student's *t*-test that compared the two averages was significant at 0.004. Subsequent *t*-tests compared the second, third, fourth, and fifth highest overlap scores between the DASSEM units with the original or randomized modules. Their p-values were also significant at 0.0029, 0.0033, 0.0036, and 0.046 respectively.

The difference in the overlap between the original versus the randomized modules shown in Figure 3 was due in part to the fact that the same proteins can be present in both the transcription modules and the DASSEM units. A statistic that compared the averages of these distributions would therefore be confounded by that fact. To assess the statistical significance of the difference in the overlap scores, the overlap of the protein content between the DASSEM units with the transcription modules versus the randomized modules was also compared, for which the randomized modules were generated by randomly redistributing the proteins across the modules. Shown in Figure 4 is a plot of the overlap scores of the DASSEM units with the original modules (black) and the randomized modules (white) using proteins as the overlap medium. A Students *t*-test that compared the averages of the distributions was significant with a p-value of 4.6*10^-3^. The test statistically validated that DASSEM units are utilized in the transcription modules. Further, the test demonstrated that DASSEM units represent a level of domain organization that can go beyond the level of protein domain architecture found with individual proteins. That is because the average overlap scores would not be different between the original versus the randomized set if the functional modules of the domain combinations did not extend beyond individual proteins. Further, subsequent Student *t*-tests that compared the second, third, fourth, and fifth DASSEM units that overlapped with the transcription modules versus the randomized modules were significant with p-values of 2.9*10^-3^, 3.4*10^-3^, 3.6*10^-3^, and 4.6*10^-2^, respectively. Since each DASSEM unit contributes a distinct function, the result validates that multiple DASSEM units can be engaged by a transcription module.

As illustrated in Figure 1, DASSEM units are formed by splicing together prevalent domain combinations that are formed by domain fusion events, and the proteins within the DASSEM units are transitively related by one or more domain. For a control analysis, the overlap scores of arbitrary groups of transitively related domains with the original versus the randomized transcription modules were compared. To generate each of these transitively related domain groups, protein pairs that had a least one domain in common were found in the *Saccharomyces cerevisiae *proteome, and all the domains within the pair were designated as a group of transitively related domains. 1,615 such groups were identified, and 114 of these were selected so that they had the same number of domains as the original DASSEM units. The overlap scores (using domains as the overlap medium) of the DASSEM units with the transcription modules was then compared to the overlap of the transitively related domains with the transcription modules. The overlap was significantly higher for the DASSEM units than the arbitrary chosen groups of transitively related domains, and that result was validated with five bootstrap samples taken, with replacement, from set of transitively related groups of domains. The result indicates that functional coherence was imparted in the DASSEM units constructed from prevalent domain combinations, and that coherence was higher than that found for arbitrary groups of transitively related domains.

**Figure 5:**
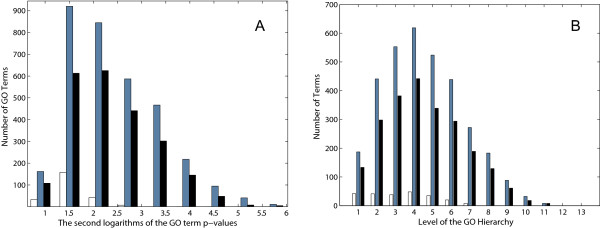
**The distributions of GO term p-values and hierarchy levels for the DASSEM units, the transcription modules, and the random protein sets**. The p-values of GO term enrichments for the DASSEM units (black), the transcription modules (gray), and the random sets of proteins (white) are shown in panel A. Since the range of p-values was large, the second logarithm, i.e. the logarithm of the absolute value of the first logarithm, was plotted for ease of visualization. The plot indicates that the number of GO terms and the values of the p-values were similar between the transcription modules and the DASSEM units. In contrast, there were much less terms associated with random sets of proteins, and the p-values of these terms were less significant. Panel B shows the levels of the GO terms within the GO hierarchy. For the transcription modules and the DASSEM units the depths of the GO term levels were similar. In contrast, the terms for the random protein sets were distributed at the higher GO levels where the terms are less specific.

**Figure 6:**
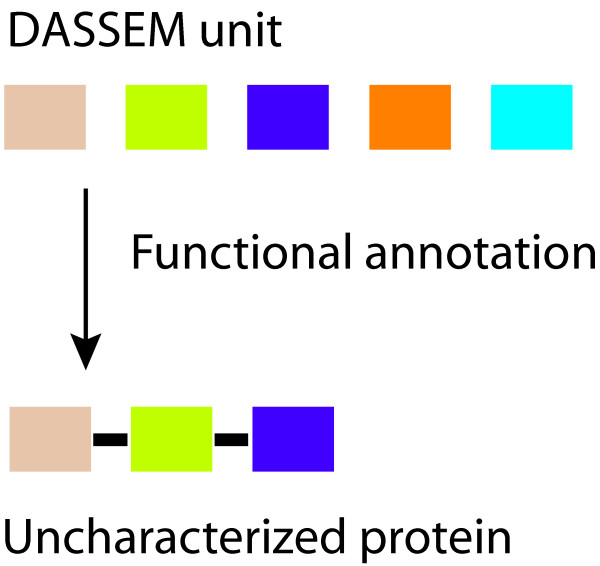
**A schematic of how the DASSEM units were used to annotate proteins of unknown function**. Domains are represented as colored blocks. If a protein of unknown function contains some of the domains of a DASSEM unit then it is likely to have all or part of the unit's function.

To further validate the functional coherence of the DASSEM units, a comparison of the GO term enrichment for the DASSEM units, the transcription modules and the randomized modules was made. The number of terms found below a p-value threshold of 0.05 with all GO term categories were considered was 2295 for the DASSEM units (median p-value 1.03*10^-4^), 3346 for the transcription modules (median p-value 9.62*10^-5^) and 240 for the randomized modules (median p-value 1.2*10^-2^). The plot shown in Figure 5 panel A shows the distributions of the p-values for the DASSEM units, the transcription modules, and the random protein sets. Similar to the transcription modules, the p-values for DASSEM units were shifted to lower (more significant) values when compared with the GO terms enriched in the random modules. The levels of the enriched GO terms within the GO hierarchy were also similar between the DASSEM units and transcription modules. The levels were deeper within the hierarchy (more specific) for both the original modules and the DASSEM units as compared with those of the randomized modules (panel B of Figure 6). The observation further confirmed that DASSEM units are functionally coherent.

### DASSEM units can be used to annotate proteins of unknown function

Given that the domains within DASSEM units function together, we deduced that would they would be useful for the annotation of uncharacterized proteins and for the prediction of new functions of proteins (See Figure 6). We reasoned that the more domains that a protein contains of a particular DASSEM unit, the more likely that it was to have the function of the unit. Among all of the proteins associated with the 114 DASSEM units identified, 393 proteins had unknown functional characteristics based on the GO annotation in SGD. We propose that the DASSEM units provide a means to assess the functional characteristic of these proteins and provide a guide for further experimental testing.

In the following we manually review five example proteins and their functional predictions. For the examples, the predictions were corroborated by evidence in the literature and by the identification of similar functional annotation for their interaction partners [58-60]. Note the interaction partners were chosen that did not contain domains of the DASSEM unit. If they did they may have been used to derive the unit; and there would be a circular argument. The analysis therefore provides independent means of verifying the predicted annotation.

One example of the use of DASSEM units for annotation is for the putative gene YBR025C. The protein product of the gene has two domains MMR_HSR1 [PFAM:PF01926] and DUF933 [PFAM:PF06071], and contributes domains to the fourth DASSEM unit listed in Table 1. That unit is annotated as having GTPase activity, being involved in the process of ribosome-nucleus export, and localizing to mitochondrion. Evidence that the YBR025C protein has these functions comes from its interaction partners Sen15, Dbp8, Rrp4 and Nup16. Sen15 is localized both to the nuclear membrane *and *to mitochondrion [61,62], Dbp8 is involved in ribosome biogenesis [63], Rrp40 functions in ribosome assembly [64], and Nup116 is a subunit of the nuclear pore complex that allows for energy-dependent rRNA export from the nucleus [65].

The protein Fun30/YAL019W contains the Helicase C domain [PFAM:PF00271] and the SNF2_N domain [PFAM:PF00176]. Its associated unit (DASSEM unit 5 in Table 1) has the function of ATPase activity, is involved in the process of chromosome organization and biogenesis, and is located in the chromatin remodeling complex. The protein has no known function based on the SGD database [42]. Evidence that Fun30 functions in the chromatin remodeling complex and has ATPase activity is that it has partial homology (35% sequence identity) to protein Snf2, which is the catalytic subunit of the chromosome remodeling complex that has ATPase activity [66,67]. An interaction partner of Fun30 is the origin replication complex ORC5, the protein complex that initiates replication and is involved in chromatin silencing [68]. Fun30 also has a genetic interaction with Swc3, a component of the chromatin remodeling complex SWR1 [67].

A third example is the protein Stb4/YMR019W, which contains the fungal specific transcription factor domain [PFAM:PF04082] and Zn(2)-Cys(6) binuclear cluster domain [PFAM:PF00172]. According to its associated DASSEM unit (the second unit in the Table 1), the protein is a putative transcriptional regulator. The GO term annotation for the process category in SGD is biological process unknown. The Stb4 protein activates transcription in a two hybrid assay without fusion to the Gal4p activation domain [69]. Its interaction partners include TAF4 [70], a subunit of the TFIID protein involved in RNA polymerase II transcription initiation [71], and Sin3 which is part of a histone deactylase complex that regulates transcription [69,72]. The evidence from the literature suggests that Stb4 is a transcription factor, which corroborates the annotation made by the DASSEM unit.

A fourth example is Dug2, which implicated in small nuclear RNA binding by a DASSEM unit. Such binding is corroborated by the fact the one of its interaction partners is Utp15, a small nuclear RNA binding protein [73,74]. The fifth example is the ORF YHL010C which, according to a DASSEM unit, is involved in ubiquitin-dependent protein catabolism. Additional evidence that the protein product of YHL010C has that function comes from the fact that it has remote homology (30% sequence identity) to the human protein Brap2, a ubiquitin E3 ligase [75].

## Discussion

From the analysis of the function linkages of DASSEM units and their utilization in transcription modules, a hierarchy of domain function was apparent. Domains combine to form functional units defined as the DASSEM units, and the DASSEM units are utilized together within transcription modules. Since the domains of a DASSEM unit can be contained within different proteins in a transcription module, DASSEM units represent a level of functional domain organization that goes beyond individual proteins. That level is necessary to provide more comprehensive functions since individual proteins are limited to contain approximately five domains [76].

The utilization of DASSEM units within transcription modules is in agreement with the results of domain fusion pair analysis [27], and extends those results. In the initial studies of domain fusion on the genomic scale [10,11], a functional linkage between two domains in separate proteins was implied when the two domains are contained within the same protein in a different species. The DASSEM units can be viewed as extended groups of domains linked by successive domain fusions that occurred throughout the evolutionary lineage of a single species. Further, Marcotte et al. deduced that one reason for domain fusion was to reduce the entropy of physical dissociation, and thereby increase a functional association [11]. In a similar way, a reduction in the entropy of physical dissociation of domains in DASSEM units may increase their functional productivity.

The domain combinations of DASSEM units are unique in different respects, and in the following some example methods for deriving domain combinations are discussed for comparison. One method is to identify domain pairs or triplets enriched across different proteins, referred to as supradomains [12]. DASSEM units correspond to some of these supradomains, but can extend the combinations to include more functionally linked domains. For example one supradomain contains the translation domain and P-loop containing nucleotide triphosphate hydrolase domain [3]. These domains correspond to the Pfam domains GTP_EFTU protein synthesis factor domain [PFAM:PF0009] and GTP_EFTU_D2 elongation factor TU domain 2 [PFAM:PF03144] that are part of the sixth DASSEM unit listed in Table 1. In total that DASSEM unit consists of seven domains, of which six are contained within the proteins IF-2, EF-TU, and EF-G. These three proteins all bind to the same site on the ribosome [77]. Also, the LepA domain recently was shown to bind to the same site on the ribosome and be involved in back translation [78]. Now all of the domains of the DASSEM unit have been demonstrated to form a functional group that bind to the same site on the ribosome. The DASSEM unit was able to find more of that functional group since it is not confined to domain combinations within individual proteins, while all the domains of supradomains must lie within an individual protein sequence.

A second method to find domain combinations is through microsyntenies of domains, i.e. their co-occurrence in small regions of the genome, found through a comparison of multiple genomes [14]. It was indicated that these combinations, referred to as domain teams, are suited for the analysis of prokaryotic genomes rather than eukaryotic genomes. Further they require multiple genomes to extract a functionally relevant group of domains.

A third method to find domain combination is through the study of domain networks. Each node in a domain network represents a domain and an edge exists if two domains co-occur within a protein [6,13]. The groups of domains represented by the DASSEM units partially coincide with some of the clusters of domains within domain networks, but there are differences due to the way they were derived. The clusters from domain networks rely on there being a relatively large clustering coefficient, i.e. dense connections between domains within the group and/or the same interaction network is present in multiple species. In contrast, the present method delineates domain combinations found within a single species and do not necessitate there being a relatively high number of links from each domain to the rest of domains in the group. An example DASSEM unit was described in Figure 1. The domain network approach does not identify the combination because there is no co-occurrence of FH and kinase domain (no edge between the two domains in the domain network) [6,13]. Further, the present method provides a flexible means of delineating a domain interaction network into functional segments. Since the present algorithm is fuzzy in the sense that the same domain can appear in different DASSEM units, it allows the network to be segmented in a way that allows the same domains to participate in different DASSEM units. That is necessary since the same domain can participate in different functions [4]. The fuzzy assignment of domains was not implemented in the domain network approach [6,13].

A concurrent study used The Discovery of All Significant Substructure (DASS) algorithm to find combinations in biological data [79]. The algorithm may be able to identify functionally linked domain combinations which are similar to DASSEM units, depending on the level of significance chosen to ensure functional linkage of all the domains in the group. Currently, the algorithm has been applied to find domain combinations containing the SH2 and PDZ domains [79].

There are a number of limitations of the current analysis. For example, the ability of DASSEM units to predict protein function of individual proteins was manual assessed and requires further validation. That validation is complicated by the fact that the function of DASSEM units can be distributed across more than one protein and may not yet be annotated at the individual protein level. In addition, for the current study a single proteome, *Saccharomyces cerevisea*, was analyzed; and that may limit the number of the functional groups identified. However, the use of data only from *S. cerevisea *ensured that relevant domain groups were found and that these groups were not contaminated by outside domains.

## Conclusion

A method was developed to identify groups of functionally linked domains. Knowledge of the groups furthers our understanding of what domains are utilized in a cooperative manner to perform a variety of cellular tasks. The groups can also provide a means to annotate uncharacterized proteins.

## Methods

### Data collection

The protein sequences and open reading frame (ORF) designations from proteins in *Saccharomyces cerevisiae *were retrieved from the UniProt database [80], which consisted of Swiss-Prot Release 47.6 and TrEMBL Release 30.6, both having the time stamp of 02-Aug-2005. These flat files were inserted into a MySQL database using a BioPerl module (Bio::SeqIO::swiss)[81]. An additional table contained the Pfam annotations downloaded from the Pfam resource [82]. An adjacency matrix, which is referred to as the *A *matrix, was then created consisting of proteins versus domains: *n *= 3,781 proteins by *m *= 1,753 domains. Within the matrix, if a protein contained one or more copies of a domain, the corresponding matrix entry was one, otherwise it was zero. Each row vector of the *A *matrix lists the domains contained by a protein and each column vector of the *A *matrix lists the proteins in which a domain is present.

### Identification of domain combinations by a SVD guided clustering method

Each domain can be considered as a vector in the protein space (each dimension is a protein) and the coordinates of a domain in this space are decided by its occurrence in each protein. A domain combination, referred to as domain assembly unit (DASSEM unit) is a cluster of domains in the protein space. Because of the large number of proteins, identification of domain clusters in such a high-dimension space is a challenge. We employed a soft-margin clustering method that was guided by singular value decomposition (SVD) [83]. SVD is a widely used spectral analysis technique to capture the significant variance of the data. The soft margin clustering method was used so that each domain was assigned, if necessary, to multiple DASSEM units to reflect the fact that a domain may participate in different domain combinations to deliver different functions.

SVD was performed on the *A *matrix using Matlab,

*A *= *USV*^*T*^,

where *U *(a *n *by *n *matrix) and *V*^*T *^(a *m *by *m *matrix, the superscript ^*T *^denotes the transposed matrix) are orthonormal, and *S *is a *n *by *m *diagonal matrix containing singular values *S*_11 _> *S*_22 _> ...... > *S*_*mm*_, *n *> *m*. Analogous to the SVD analyses on gene expression microarray data [84,85], the *i*th row of *V*^*T*^, viT⇀
 MathType@MTEF@5@5@+=feaafiart1ev1aaatCvAUfKttLearuWrP9MDH5MBPbIqV92AaeXatLxBI9gBaebbnrfifHhDYfgasaacH8akY=wiFfYdH8Gipec8Eeeu0xXdbba9frFj0=OqFfea0dXdd9vqai=hGuQ8kuc9pgc9s8qqaq=dirpe0xb9q8qiLsFr0=vr0=vr0dc8meaabaqaciaacaGaaeqabaqabeGadaaakeaadaWhkaqaaiabdAha2naaDaaaleaacqWGPbqAaeaacqWGubavaaaakiaawgniaaaa@329A@, is a linear combination of protein vectors (rows of *A *matrix) and is called eigenprotein. The *j*th element of viT⇀
 MathType@MTEF@5@5@+=feaafiart1ev1aaatCvAUfKttLearuWrP9MDH5MBPbIqV92AaeXatLxBI9gBaebbnrfifHhDYfgasaacH8akY=wiFfYdH8Gipec8Eeeu0xXdbba9frFj0=OqFfea0dXdd9vqai=hGuQ8kuc9pgc9s8qqaq=dirpe0xb9q8qiLsFr0=vr0=vr0dc8meaabaqaciaacaGaaeqabaqabeGadaaakeaadaWhkaqaaiabdAha2naaDaaaleaacqWGPbqAaeaacqWGubavaaaakiaawgniaaaa@329A@, vijT
 MathType@MTEF@5@5@+=feaafiart1ev1aaatCvAUfKttLearuWrP9MDH5MBPbIqV92AaeXatLxBI9gBaebbnrfifHhDYfgasaacH8akY=wiFfYdH8Gipec8Eeeu0xXdbba9frFj0=OqFfea0dXdd9vqai=hGuQ8kuc9pgc9s8qqaq=dirpe0xb9q8qiLsFr0=vr0=vr0dc8meaabaqaciaacaGaaeqabaqabeGadaaakeaacqWG2bGDdaqhaaWcbaGaemyAaKMaemOAaOgabaGaemivaqfaaaaa@3237@, is the coordinate of the *j*th domain of the eigenprotein viT⇀
 MathType@MTEF@5@5@+=feaafiart1ev1aaatCvAUfKttLearuWrP9MDH5MBPbIqV92AaeXatLxBI9gBaebbnrfifHhDYfgasaacH8akY=wiFfYdH8Gipec8Eeeu0xXdbba9frFj0=OqFfea0dXdd9vqai=hGuQ8kuc9pgc9s8qqaq=dirpe0xb9q8qiLsFr0=vr0=vr0dc8meaabaqaciaacaGaaeqabaqabeGadaaakeaadaWhkaqaaiabdAha2naaDaaaleaacqWGPbqAaeaacqWGubavaaaakiaawgniaaaa@329A@ in the eigenprotein space. Correspondingly, the *j*th column of *U*, uj⇀
 MathType@MTEF@5@5@+=feaafiart1ev1aaatCvAUfKttLearuWrP9MDH5MBPbIqV92AaeXatLxBI9gBaebbnrfifHhDYfgasaacH8akY=wiFfYdH8Gipec8Eeeu0xXdbba9frFj0=OqFfea0dXdd9vqai=hGuQ8kuc9pgc9s8qqaq=dirpe0xb9q8qiLsFr0=vr0=vr0dc8meaabaqaciaacaGaaeqabaqabeGadaaakeaadaWhkaqaaiabdwha1naaBaaaleaacqWGQbGAaeqaaaGccaGLrdcaaaa@3168@, is a linear combination of all domain vectors (columns of *A *matrix) and is called eigendomain. The *i*th element of uj⇀
 MathType@MTEF@5@5@+=feaafiart1ev1aaatCvAUfKttLearuWrP9MDH5MBPbIqV92AaeXatLxBI9gBaebbnrfifHhDYfgasaacH8akY=wiFfYdH8Gipec8Eeeu0xXdbba9frFj0=OqFfea0dXdd9vqai=hGuQ8kuc9pgc9s8qqaq=dirpe0xb9q8qiLsFr0=vr0=vr0dc8meaabaqaciaacaGaaeqabaqabeGadaaakeaadaWhkaqaaiabdwha1naaBaaaleaacqWGQbGAaeqaaaGccaGLrdcaaaa@3168@, *u*_*ij*_, is the coordinate of the *i*th protein of the eigendomain uj⇀
 MathType@MTEF@5@5@+=feaafiart1ev1aaatCvAUfKttLearuWrP9MDH5MBPbIqV92AaeXatLxBI9gBaebbnrfifHhDYfgasaacH8akY=wiFfYdH8Gipec8Eeeu0xXdbba9frFj0=OqFfea0dXdd9vqai=hGuQ8kuc9pgc9s8qqaq=dirpe0xb9q8qiLsFr0=vr0=vr0dc8meaabaqaciaacaGaaeqabaqabeGadaaakeaadaWhkaqaaiabdwha1naaBaaaleaacqWGQbGAaeqaaaGccaGLrdcaaaa@3168@ in the eigendomain space. Since there are much more proteins than domains, we chose to cluster proteins in the eigendomain space and then found the corresponding domain clusters because this procedure is more robust than clustering domains directly in the eigenprotein space.

The proteins were clustered in the eigendomain space using the K-means algorithm, and a mixture Gaussian model was built upon these initial clusters in order to assign proteins to multiple clusters [86]. For the K-means step, we initialized the centroids of clusters as the following. Proteins were first clustered using only the first two eigendomains, u1⇀
 MathType@MTEF@5@5@+=feaafiart1ev1aaatCvAUfKttLearuWrP9MDH5MBPbIqV92AaeXatLxBI9gBaebbnrfifHhDYfgasaacH8akY=wiFfYdH8Gipec8Eeeu0xXdbba9frFj0=OqFfea0dXdd9vqai=hGuQ8kuc9pgc9s8qqaq=dirpe0xb9q8qiLsFr0=vr0=vr0dc8meaabaqaciaacaGaaeqabaqabeGadaaakeaadaWhkaqaaiabdwha1naaBaaaleaacqaIXaqmaeqaaaGccaGLrdcaaaa@30FB@ and u2⇀
 MathType@MTEF@5@5@+=feaafiart1ev1aaatCvAUfKttLearuWrP9MDH5MBPbIqV92AaeXatLxBI9gBaebbnrfifHhDYfgasaacH8akY=wiFfYdH8Gipec8Eeeu0xXdbba9frFj0=OqFfea0dXdd9vqai=hGuQ8kuc9pgc9s8qqaq=dirpe0xb9q8qiLsFr0=vr0=vr0dc8meaabaqaciaacaGaaeqabaqabeGadaaakeaadaWhkaqaaiabdwha1naaBaaaleaacqaIYaGmaeqaaaGccaGLrdcaaaa@30FD@. The initial centroids were randomly generated. The number of clusters *K *was adjusted so that the *K *giving the maximum Calinski-Harabasz (CH) index, a quantity which describes the degree of inter-cluster separation and intra-cluster homogeneity [87,88]. The obtained centroids and the optimal number of clusters were used as the initial parameters for clustering the proteins in the space of u1⇀
 MathType@MTEF@5@5@+=feaafiart1ev1aaatCvAUfKttLearuWrP9MDH5MBPbIqV92AaeXatLxBI9gBaebbnrfifHhDYfgasaacH8akY=wiFfYdH8Gipec8Eeeu0xXdbba9frFj0=OqFfea0dXdd9vqai=hGuQ8kuc9pgc9s8qqaq=dirpe0xb9q8qiLsFr0=vr0=vr0dc8meaabaqaciaacaGaaeqabaqabeGadaaakeaadaWhkaqaaiabdwha1naaBaaaleaacqaIXaqmaeqaaaGccaGLrdcaaaa@30FB@, u2⇀
 MathType@MTEF@5@5@+=feaafiart1ev1aaatCvAUfKttLearuWrP9MDH5MBPbIqV92AaeXatLxBI9gBaebbnrfifHhDYfgasaacH8akY=wiFfYdH8Gipec8Eeeu0xXdbba9frFj0=OqFfea0dXdd9vqai=hGuQ8kuc9pgc9s8qqaq=dirpe0xb9q8qiLsFr0=vr0=vr0dc8meaabaqaciaacaGaaeqabaqabeGadaaakeaadaWhkaqaaiabdwha1naaBaaaleaacqaIYaGmaeqaaaGccaGLrdcaaaa@30FD@ and u3⇀
 MathType@MTEF@5@5@+=feaafiart1ev1aaatCvAUfKttLearuWrP9MDH5MBPbIqV92AaeXatLxBI9gBaebbnrfifHhDYfgasaacH8akY=wiFfYdH8Gipec8Eeeu0xXdbba9frFj0=OqFfea0dXdd9vqai=hGuQ8kuc9pgc9s8qqaq=dirpe0xb9q8qiLsFr0=vr0=vr0dc8meaabaqaciaacaGaaeqabaqabeGadaaakeaadaWhkaqaaiabdwha1naaBaaaleaacqaIZaWmaeqaaaGccaGLrdcaaaa@30FF@. The procedure was iterated until 135 eigendomains were used and 114 clusters were found. Note that we selected the top 135 eigendomains for the clustering as the 135^th ^singular value was already quite small, and subsequent clusters became unstable between the K-means and mixture Gaussian fitting steps, with some clusters being produced with zero content.

After the K-means clustering step, each protein was assigned to only one cluster. Since domains may participate in multiple combinations, it was important to allow flexible assignment of proteins, and their corresponding domains, to multiple clusters. We modeled each cluster obtained from K-means using a Gaussian distribution, centered at the centroid of the cluster. The variance of each Gaussian distribution was determined by maximizing the likelihood of the data,

∏i=1K∏j=1n12πσiexp⁡[−(xj⇀−x⇀i0)2σi2],
 MathType@MTEF@5@5@+=feaafiart1ev1aaatCvAUfKttLearuWrP9MDH5MBPbIqV92AaeXatLxBI9gBaebbnrfifHhDYfgasaacH8akY=wiFfYdH8Gipec8Eeeu0xXdbba9frFj0=OqFfea0dXdd9vqai=hGuQ8kuc9pgc9s8qqaq=dirpe0xb9q8qiLsFr0=vr0=vr0dc8meaabaqaciaacaGaaeqabaqabeGadaaakeaadaqeWbqaamaarahabaWaaSaaaeaacqaIXaqmaeaadaGcaaqaaiabikdaYGGaciab=b8aWbWcbeaakiab=n8aZnaaBaaaleaacqWGPbqAaeqaaaaaaeaacqWGQbGAcqGH9aqpcqaIXaqmaeaacqWGUbGBa0Gaey4dIunaaSqaaiabdMgaPjabg2da9iabigdaXaqaaiabdUealbqdcqGHpis1aOGagiyzauMaeiiEaGNaeiiCaa3aamWaaeaacqGHsisldaWcaaqaamaabmaabaWaa8HcaeaacqWG4baEdaWgaaWcbaGaemOAaOgabeaaaOGaayz0GaGaeyOeI0Yaa8HcaeaacqWG4baEaiaawgniamaaDaaaleaacqWGPbqAaeaacqaIWaamaaaakiaawIcacaGLPaaaaeaacqaIYaGmcqWFdpWCdaqhaaWcbaGaemyAaKgabaGaeGOmaidaaaaaaOGaay5waiaaw2faaiabcYcaSaaa@5B73@

where *K *is the number of clusters, *n *is the number of proteins considered in the analysis, xj⇀
 MathType@MTEF@5@5@+=feaafiart1ev1aaatCvAUfKttLearuWrP9MDH5MBPbIqV92AaeXatLxBI9gBaebbnrfifHhDYfgasaacH8akY=wiFfYdH8Gipec8Eeeu0xXdbba9frFj0=OqFfea0dXdd9vqai=hGuQ8kuc9pgc9s8qqaq=dirpe0xb9q8qiLsFr0=vr0=vr0dc8meaabaqaciaacaGaaeqabaqabeGadaaakeaadaWhkaqaaiabdIha4naaBaaaleaacqWGQbGAaeqaaaGccaGLrdcaaaa@316E@ is the coordinate vector of the *j*th protein in the eigendomain space, x⇀i0
 MathType@MTEF@5@5@+=feaafiart1ev1aaatCvAUfKttLearuWrP9MDH5MBPbIqV92AaeXatLxBI9gBaebbnrfifHhDYfgasaacH8akY=wiFfYdH8Gipec8Eeeu0xXdbba9frFj0=OqFfea0dXdd9vqai=hGuQ8kuc9pgc9s8qqaq=dirpe0xb9q8qiLsFr0=vr0=vr0dc8meaabaqaciaacaGaaeqabaqabeGadaaakeaadaWhkaqaaiabdIha4bGaayz0GaWaa0baaSqaaiabdMgaPbqaaiabicdaWaaaaaa@3251@ and *σ*_*i *_are the mean and variance of the *i*th Gaussian distribution respectively. The final assignment of a protein to a given cluster was based on a threshold level of probability, set to be within three standard deviations of the mean of the cluster. A protein could thereby be assigned to multiple clusters. The domain groups associated with the protein clusters were found using the relation *V *= *A*^*T*^*US*^-1^. These domain groups were referred to as the domain assembly units (DASSEM units). Functional annotation of each DASSEM unit was found by applying the GO::TermFinder tool to the list of proteins associated with the unit [32]. To correct for multiple hypothesis testing, the Bonferroni correction was used when GO term enrichment p-values were calculated [34].

### Overlap between DASSEM units and transcription modules

To show how DASSEM units were utilized, the content of the units was compared to that found in groups of proteins in transcription modules. From a study by Imhels et al., 86 transcription modules were obtained [55,56]. An overlap score, OS, was calculated for each DASSEM unit that contained domains overlapped with a given transcription module. The overlap score was the fraction of domains within a DASSEM unit that are in common with the transcription module multiplied by the fraction of domains within the transcription module that are in common with the DASSEM unit:

OS=NcNm×NcNd,
 MathType@MTEF@5@5@+=feaafiart1ev1aaatCvAUfKttLearuWrP9MDH5MBPbIqV92AaeXatLxBI9gBaebbnrfifHhDYfgasaacH8akY=wiFfYdH8Gipec8Eeeu0xXdbba9frFj0=OqFfea0dXdd9vqai=hGuQ8kuc9pgc9s8qqaq=dirpe0xb9q8qiLsFr0=vr0=vr0dc8meaabaqaciaacaGaaeqabaqabeGadaaakeaacqWGpbWtcqWGtbWucqGH9aqpdaWcaaqaaiabd6eaonaaBaaaleaacqWGJbWyaeqaaaGcbaGaemOta40aaSbaaSqaaiabd2gaTbqabaaaaOGaey41aq7aaSaaaeaacqWGobGtdaWgaaWcbaGaem4yamgabeaaaOqaaiabd6eaonaaBaaaleaacqWGKbazaeqaaaaakiabcYcaSaaa@3DDD@

where *N*_*c *_is the number of domains that are in common between the DASSEM unit and the module, *N*_*M *_is the total number of domains in the module, and *N*_*D *_is the number of domains in the DASSEM unit. The overlap score was used to provide an overall measure the degree of functional utilization of a DASSEM unit within a transcription module. It considers the degree to which the function of the DASSEM unit is utilized and the degree to which the function of the unit contributes to the module.

DASSEM units were ranked according to their overlap scores for each module. From the highest ranking DASSEM units, four DASSEM unit collections were generated for each module by combining the domains in units ranked one and two, units ranked one through three, units ranked one through four, and units ranked one through five. The cumulative overlap score of each collection with each module was calculated. The process of finding overlapping DASSEM units and collections was done for each of the 86 co-expression modules.

A randomization protocol was employed to compare the average overlap scores for the highest overlapping DASSEM units with the original modules versus random sets of proteins. The random sets of proteins were created by randomly redistributing the proteins among the transcription modules while keeping the number of proteins in each module constant. Since the proteins were randomized, the domains within a given protein remained together. Statistical significance of the overlap scores were estimated by the p-values of Student's *t*-tests comparing the average overlap scores of DASSEM units with the original transcription modules versus the random sets of proteins. The other control analyses were fully explained in the results section.

## Authors' contributions

WAM participated in the design of the study, performed data analysis, interpreted the results and wrote the manuscript. KC participated in the design of the study and wrote the Matlab computer code used for clustering. TH participated in the design of the study and performed data analysis to test the utility of the clustering algorithm. WW participated in the design of the study, interpreted the results and wrote the manuscript.

All authors read and approved the final manuscript.
